# Brugada Phenocopy Induced by Hypovolemic Hyponatremia

**DOI:** 10.7759/cureus.45667

**Published:** 2023-09-21

**Authors:** Emre Yılmaz, Fatih Özdemir

**Affiliations:** 1 Cardiology, Giresun University, Faculty of Medicine, Giresun, TUR

**Keywords:** brugada electrocardiogram pattern, brugada phenocopy, brugada-like pattern, hypovolemic hyponatremia, brugada syndrome

## Abstract

Brugada syndrome (BrS) is a hereditary channelopathy caused by an autosomal dominant mutation in the cardiac sodium channel gene SCN5A alpha subunit. In individuals without structural heart disease, the risk of sudden cardiac death (SCD) increases in this channelopathy with ST-segment elevation in V1-3 precordials. Brugada phenocopy (BrP) is a condition in which transient ST-segment elevations are observed, mimicking BrS electrocardiographic changes, which can occur with electrolyte and metabolic disorder scenarios. In this study, we share a case of BrP that occurred due to hypovolemic hyponatremia and recovered spontaneously with the correction of electrolyte disturbance.

## Introduction

Brugada syndrome (BrS) is an autosomal dominant inherited channelopathy with pseudo-right bundle branch block on electrocardiography (ECG) and persistent ST segment elevations, especially in leads V1-2, with an increased risk of ventricular tachyarrhythmia (VT) and sudden cardiac death (SCD) [1-4]. Transient ST segment elevations, defined as Brugada phenocopy (BrP), may be observed in some individuals whose BrS mimics ECG findings but without a family history of SCD, syncope, or VT. These ECG changes have been shown in metabolic and electrolyte disorders, especially hyperkalemia and hyponatremia, in clinical situations such as ischemia, acidosis, and hypothyroidism. Spontaneous regression of ST segment elevations with improving the underlying clinical disorder is essential in distinguishing BrP from BrS [5]. BrP refers to patients presenting to emergency departments with underlying noisy clinical scenarios and can be confused with ST-segment elevation myocardial infarction. We hope that case examples in different clinical scenarios will raise clinicians' awareness of the appropriate management of the patient group with BrP, who may require a rapid treatment plan for the underlying pathology. In this study, we share a case of BrP caused by hypovolemic hyponatremia.

## Case presentation

An 84-year-old male patient was brought to the Emergency Department at Giresun University, Faculty of Medicine, complaining of weakness, loss of appetite, nausea, and sleepiness for the past week. His Glasgow Coma Scale (GCS) was 13. The patient was treated for hypertension, dementia, benign prostatic hyperplasia, and chronic obstructive pulmonary disease. And he had no history of cardiac disease. We learned that the patient, who has been immobile for five years, had diarrhea 10 days ago and his nutrition had decreased in the last week, and his complaints started. He had confusion, drowsiness, and speech disorder. There was no sign of lateralization. There was soft character edema in bilateral extremities (+/+) and sacrum. Skin turgor and tone were decreased. His skin and tongue were dry. We found no pathological features in other system examinations. Body mass index was 25.2 kg/m^2^, temperature was 37.7°C, heart rate was 102 beats/min, oxygen saturation measured with a pulse oximeter was 96% in room air, and blood pressure was 100/70 mmHg. Medications that the patient used regularly are as follows: donezepil hydrochloride + memantine hydrochloride 5/20 mg 1×1, ipratropium bromide monohydrate + salbutamol sulfate 0.5/2.5 mg 4×1, thioctic acid 600 mg 1×1, sodium valproate 500 mg 2×1, dutasteride 0.5 mg 1×1, and carvedilol 12.5 mg 2×1. We presented the laboratory results at first admission and discharge in Table 1. At the patient's emergency admission, blood sugar was normal. The creatinine value was compatible with chronic kidney disease. Other laboratory tests and electrolyte levels other than sodium were within normal limits. Initial laboratory results indicated a serum sodium level of 109 mEq/L (normal range: 135-145 mEq/L). His serum osmolality was 246 mOsm/kg (normal range: 275-295 mOsm/kg), and urinary osmolality was 586 mOsm/kg (normal range: 300-900 mOsm/kg). A random urinary sodium was 20 mEq/L. The patient, whom we categorized as chronic severe hyponatremia since the onset of symptoms was longer than 48 hours, presented with clinically moderate to severe symptoms.

**Table 1:** Laboratory results at first admission and discharge.

Variables	Reference ranges	At first admission	Discharge
Glucose	75-99 mg/dL	101 mg/dL	110 mg/dL
Urea	16.6-48.5 mg/dL	108 mg/dL	104 mg/dL
Creatinine	0.7-1.20 mg/dL	1.48 mg/dL	1.39 mg/dL
Glomerular filtration rate	>90 mL/dk	45.95 mL/dk	49.57 mL/dk
Alanine aminotransferase	10-41 U/L	13 U/L	11 U/L
Aspartate aminotransferase	0-40 U/L	19 U/L	16 U/L
Albumin	3.4-5.4 g/dL	3.59 g/dL	3.61 g/dL
Total bilirubin	0-1.2 mg/dL	0.34 mg/dL	0.22 mg/dL
Direct bilirubin	0-0.3 mg/dL	0.09 mg/dL	0.1 mg/dL
Creatine kinase	<190 U/L	47 U/L	15 U/L
Lactate dehydrogenase	135-225 U/L	148 U/L	123 U/L
pH	7.35-7.45	7.38	7.41
Calcium	8.8-10.2 mg/dL	9.5 mg/dL	9.4 mg/dL
Sodium	136-145 mEq/L	109 mEq/L	136 mEq/L
Chlorine	98-107 mEq/L	98 mEq/L	102 mEq/L
Potassium	3.5-5.1 mEq/L	4.1 mEq/L	4.2 mEq/L
C-reactive protein	<5.0 mg/L	5.57 mg/L	4.43 mg/L
White blood cell	4-11×10^9^/L	10×10^9^/L	8.56×10^9^/L
Hemoglobin	13.5-17.5 g/dL	10.5 g/dL	11 g/dL
Mean corpuscular volume	80-96 fL	99.4 fL	98.6 fL
Platelets	150-450×10^9^/L	159×10^9^/L	156×10^9^/L
Neutrophil	2-7×10^9^/L	10.05×10^9^/L	6.65×10^9^/L
Lymphocyte	0.8-4×10^9^/L	0.72×10^9^/L	0.75×10^9^/L

**Figure 1:**
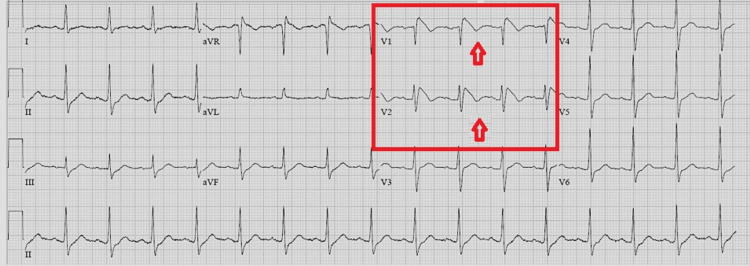
Electrocardiogram showing Brugada type I pattern in V1 and V2 leads (red arrows).

The patient with impaired consciousness was first evaluated in terms of neurological pathologies. Brain computed tomography and diffusion magnetic resonance imaging were normal. We hospitalized the patient diagnosed with hypovolemic hyponatremia. And we treated him with recurrent 3% saline. Saline administration was as follows: it was started with a 1 mL/kg 3% saline bolus (maximum 100 mL) and continued with a 15-30 mL/h infusion protocol after the 6th hour. The basic rhythm of the patient's ECG was normal sinus rhythm. Since coved-type ST-segment elevation was observed in leads V1 and V2, the patient was consulted by our cardiology unit (Figure 1).

The patient had no anginal complaint in the emergency room presentation or history. There was no previous history of cardiac disease. According to relatives upon admission, there was no pertinent history of syncope, presyncope palpitations, or family history of SCD. The echocardiographic evaluation revealed an ejection fraction of 65%, mild mitral regurgitation, and mild tricuspid regurgitation. Measurements of heart chambers were normal. No wall motion defect was detected.

**Figure 2:**
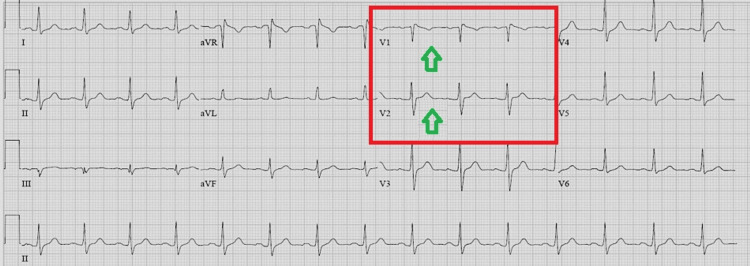
Electrocardiogram showing the resolution of Brugada type I pattern (green arrows).

The patient's troponin levels at admission, first hour, and fourth hour were normal. We accepted the first admission ECG of the patient as a Brugada type 1 pattern. We did not plan the primary percutaneous coronary intervention. We followed the patient with ECG and troponin monitoring. On the fourth follow-up day, the patient's sodium level returned to normal range, and his consciousness and speech disorder completely recovered. On the control ECG of the fourth follow-up day, we found the ST segment elevation in V1 and V2 regressed (Figure 2). Unfortunately, the patient did not consent to an ajmaline provocation test, electrophysiological studies, or signal-averaged ECG tests for advanced diagnosis and risk assessment. This ECG pattern we detected was evaluated as a BrP due to hypovolemic hyponatremia. No ventricular/atrial dysrhythmias and no troponin increase were observed in the patient's cardiac monitoring during the hospital stay.

## Discussion

Brugada syndrome (BrS) is a hereditary channelopathy transmitted by autosomal dominant inheritance, increasing arrhythmogenic susceptibility. The risk of VT and SCD increases in this disease that typically produces pseudo-right bundle branch block on ECG and persistent ST-segment elevation, particularly in leads V1-2. It has been reported that similar ECG findings can also be observed in inferior ECG leads in some isolated cases [1-4,6]. These ECG changes typical of BrS may be encountered in some special patient groups without syncope attacks, VT, or SCD. This pattern, which can be confused with ST-segment elevation myocardial infarction, is called BrP. BrP can be observed in metabolic disorders based on electrolyte imbalance and clinical conditions such as acidosis, ischemia, hypothyroidism, and right ventricular compression [5].

**Figure 3:**
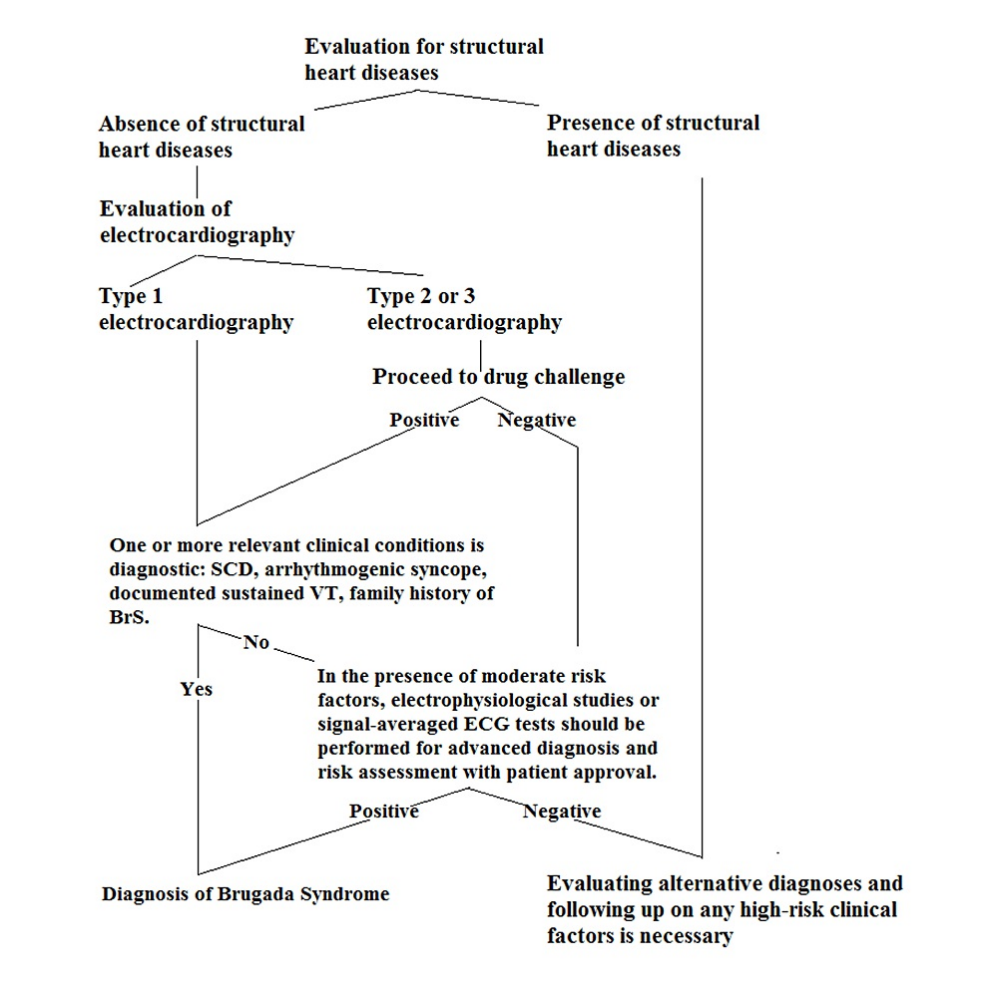
Brugada syndrome diagnostic approach algorithm.

We show the diagnostic approach algorithm of patients with moderate and high suspicion for BrS in Figure 3. Structural cardiac pathologies should be evaluated first in BrS and BrP. Structural cardiac pathologies lead us to alternative diagnoses, but close follow-up of patients is still essential in the presence of high-risk clinical factors. Type 1 ECG pattern is an important diagnostic factor. Another essential diagnostic factor is clinical factors that create high risk. These are SCD, arrhythmogenic syncope, documented sustained VT, and family history of BrS. Clinicians should meticulously question these factors in the patient's anamnesis, history, and family history. Type 2 and 3 ECG patterns differ with minor nuances, so the diagnostic algorithms are similar. Applying a drug challenge test to patients with type 2 and 3 ECG patterns is necessary. Electrophysiological studies and signal-averaged ECG tests are other diagnostic tools that provide valuable information for further investigation and risk stratification in diagnosing BrS.

BrS characteristics of ST segment abnormalities in leads V1 to V3 are as follows: (i) type 1 - J wave amplitude is 2 mm and above, the T wave is negative, and ST-T configuration is coved type. The ST segment terminal portion tends to descend gradually. (ii) Types 2 and 3 - J wave amplitude is also ≥2 mm. While the T wave is positive or biphasic in type 2, it is positive in type 3. ST-T configuration is saddle-back type in both. ST-segment terminal portion elevated ≥1 mm in type 2 and ≤1 mm in type 3 [7].

In BrS, the ECG has two important ST segment patterns, especially in leads V1-V2. These are the coved and saddle-back patterns. In the coved pattern, the ST segment is observed at the end of the QRS, rising with a high take-off of ≥2 mm and descending with a rapid slope at a concave or eastern angle. There is no clear r' wave. The coved pattern high take-off often does not correspond to the J point, and the ST segment's amplitude reduction is 4 mm or less within 40 ms of the high take-off. A negative and symmetrical T wave follows the ST segment. In the coved pattern, the duration of the QRS is longer than that measured in the right bundle branch block, and there may be discrepancies between V1 and V6. In the saddle-back pattern, the r' high take-off is 2 mm and above, although it does not usually overlap with the J point. The descending arm r' coincides with the beginning of the ST segment, although it is not always well visible. ST height is at least 0.5 mm and above. In the saddle-back pattern, the ST segment is followed by a positive T wave in V2, although it has variable morphology in V1. The triangle formed by the ascending and descending arms of r' offers different diagnostic criteria with factors such as the β angle and the duration of the triangular base. QRS duration is longer than in cases with r' in V1, and there may be discrepancies between leads V1-V6 [8].

The Brugada pattern on the ECG can be seen as an early subclinical manifestation of arrhythmogenic right ventricular cardiomyopathy (ARVC) [9]. ARVC is an autosomal dominant disease that primarily affects the right ventricle. It is a hereditary myopathy where the right ventricular myocardium is replaced by adipose tissue, usually with diffuse myocardial cells and fibrous tissue. Patients with ARVC often have abnormalities of the right ventricle that can be seen on echocardiography or cardiac magnetic resonance imaging. In contrast, most BrS patients do not show significant structural heart disease on routine imaging studies. Other pathologies that can cause ST segment change in leads V1-2 are right bundle branch block, early repolarization, acute pericarditis, acute myocardial ischemia or infarction, left ventricular hypertrophy, pectus excavatum, hypothermia, and blunt chest trauma [10].

Some drugs may cause a Brugada-like pattern on ECG. As does the febrile state, these drugs cause an existing BrS to come to light. If we summarize these drugs under the main headings, antiarrhythmic or antianginal drugs, psychotropic drugs, alcohol intoxication, cocaine intoxication, and dimenhydrinate can be counted. Some of the cardiac sodium channel blockers from antiarrhythmic or antianginal drugs are used for drug challenges in patients with Brugada types 2 and 3. These drugs are flecainide and propafenone from the class IC group and ajmaline and procainamide from the class IA group. Pilsicainide from the class IC group, disopyramide, and cibenzoline from the class IA group are other antiarrhythmic drugs that are not used in drug challenges but can have Brugada-like patterns. It should not be forgotten that calcium channel blockers, beta-blockers, nitrates, and nicorandil (a potassium channel opener), frequently preferred in cardiology clinical practice, can also create a Brugada-like EKG pattern [10]. Our patient was also using beta-blockers as antihypertensive. However, this treatment was not interrupted because the current clinical and laboratory findings suggest hyponatremia is the primary suspect condition. ST-segment elevation, which spontaneously improved with the correction of electrolyte disturbance, distracted us from the idea of a beta-blocker-related Brugada-like pattern.

Mutations detected in SCN5A and SCN10A of the SCN gene family, which encode the subunits of cardiac sodium channels, have been implicated in the BrS's genetic background. This genetic defect in cardiac sodium channels results in decreased sodium input currents and shortened action potential duration. Defective cardiac sodium channel dysfunction most commonly affects the right ventricular outflow tract (RVOT). A distinct transient outward current in the RVOT epicardium called "Ito" results in a marked shortening of the action potential when sodium input is reduced [11]. Noninvasive ECG mappings show that the transmural voltage gradient caused by the repolarization disorder in RVOT causes ST-segment elevation reflected in the ECG. The same studies additionally showed evidence of an arrhythmogenic substrate in RVOT with delayed activation, slow conduction, and steep repolarization gradients between RVOT and the rest of the right ventricle [12].

Hyponatremia, defined as a serum sodium concentration below 135 mEq/L, may occur due to a single cause, or it may be associated with multiple factors and is usually caused by the inability to excrete water normally [13]. Hyponatremia causes a decrease in sodium ion current in the cell due to the decreasing sodium gradient. Decreased sodium ion transport may contribute to the inability to oppose outward potassium ion channels. This resulting imbalance of ion transport may also contribute to the Brugad-like ECG pattern [11,14].

There are examples of BrP presenting with different clinical scenarios in the literature. In their article, where they shared a case of BrP due to hyponatremia and hyperkalemia associated with postrenal renal failure after prostate surgery, the authors reported that eliminating electrolyte disturbance with urinary catheterization and replacement therapy improved ECG findings quickly [15]. Landa et al. reported a demonstrative type 1 Brugada pattern presenting with diabetic ketoacidosis, hyponatremia, and hyperkalemia in a follow-up patient diagnosed with type 1 diabetes mellitus. In their presentation, the authors demonstrated that the elimination of metabolic and electrolyte disturbances produced a dramatic improvement in ECG findings [16]. Multiple electrolyte disturbances are not always necessary for BrP. As in our case, an isolated single electrolyte disorder may also cause typical ECG findings. Ramsaroop et al. reported hypovolemic hyponatremia associated with hydrochlorothiazide and angiotensin-converting enzyme inhibitors and associated Brugada type 1 ST-segment elevation in a 49-year-old hypertensive patient. Changing his antihypertensive treatment and correcting the electrolyte disturbance improved the patient's ST-segment elevation [17]. On the other hand, it should be remembered that multiple electrolyte disturbances associated with primary adrenal insufficiency and hyponatremia due to excessive water and alcohol consumption may also present with BrP [18,19]. Even in all these scenarios, eliminating metabolic and electrolyte disorders resulted in the recovery of spontaneous ST-segment elevation. In our case, we presented a BrP scenario caused by hypovolemic hyponatremia secondary to gastrointestinal loss and oral intake disorder for the clinicians' discretion. Even in this scenario, we wanted to draw attention to the importance of ECG findings and history, which reached a significant level in emergency service admissions in elderly patients. The BrP case we present is suitable for type 1 BrP class B according to the classification by Gottschalk et al. [20]. Eliminating our patient's electrolyte disturbance improved ST-segment elevation and impaired consciousness associated with hyponatremia, consistent with other examples in the literature.

BrS is a hereditary disease that can progress with fatal arrhythmias. It is a subject that has been researched for many years, and diagnostic and treatment modalities have been developed. However, BrP remains an intriguing mystery. We shared this case in the hope that BrP cases occurring in different clinical scenarios will raise awareness in clinicians about diagnosis and follow-up.

## Conclusions

BrP is a condition that can be easily confused with ST-elevation myocardial infarction, may cause patients to undergo inappropriate tests and treatments, and, most importantly, may delay the appropriate treatment for the underlying disease. In this patient group, it is essential to evaluate the ECG changes with a meticulous history, symptoms, findings, and laboratory results. ST-segment changes in BrP that occur due to electrolyte disturbance resolve spontaneously with correction of the underlying electrolyte disorder. We presented such a case to help physicians understand this phenomenon and its presentation.
